# Identification of Quantitative Trait Loci That Determine Plasma Total-Cholesterol and Triglyceride Concentrations in DDD/Sgn and C57BL/6J Inbred Mice

**DOI:** 10.1155/2017/3178204

**Published:** 2017-05-31

**Authors:** Jun-ichi Suto, Misaki Kojima

**Affiliations:** ^1^Institute of Agrobiological Sciences, National Agriculture and Food Research Organization (NARO), Tsukuba, Ibaraki 305-8634, Japan; ^2^Institute of Livestock and Grassland Science, National Agriculture and Food Research Organization (NARO), Tsukuba, Ibaraki 305-0901, Japan

## Abstract

DDD/Sgn mice have significantly higher plasma lipid concentrations than C57BL/6J mice. In the present study, we performed quantitative trait loci (QTL) mapping for plasma total-cholesterol (CHO) and triglyceride (TG) concentrations in reciprocal F_2_ male intercross populations between the two strains. By single-QTL scans, we identified four significant QTL on chromosomes (Chrs) 1, 5, 17, and 19 for CHO and two significant QTL on Chrs 1 and 12 for TG. By including cross direction as an interactive covariate, we identified separate significant QTL on Chr 17 for CHO but none for TG. When the large phenotypic effect of QTL on Chr 1 was controlled by composite interval mapping, we identified three additional significant QTL on Chrs 3, 4, and 9 for CHO but none for TG. QTL on Chr 19 was a novel QTL for CHO and the allelic effect of this QTL significantly differed between males and females. Whole-exome sequence analysis in DDD/Sgn mice suggested that* Apoa2* and* Acads* were the plausible candidate genes underlying CHO QTL on Chrs 1 and 5, respectively. Thus, we identified a multifactorial basis for plasma lipid concentrations in male mice. These findings will provide insight into the genetic mechanisms of plasma lipid metabolism.

## 1. Introduction

Plasma lipid concentrations are representative quantitative traits; that is, they are controlled by multiple genes under the influence of nonheritable environmental effects. Among plasma lipids, cholesterol (CHO) and triglyceride (TG) have clinical implications in atherosclerosis and coronary artery disease; therefore, it is crucially important to identify genes influencing variations in plasma CHO and TG concentrations [1, 2] (in this manuscript, we use the term “plasma lipid” when CHO and TG are simultaneously referred to). Many studies have aimed to dissect the genetic bases underlying plasma lipid concentrations in mice, and more than one hundred CHO QTL and more than fifty TG QTL have been reported to date ([3, 4] and Mouse Genome Informatics (MGI, http://www.informatics.jax.org/)). Most QTL identified in mice have conserved synteny with human loci that control plasma lipid concentrations and diseases [5].

We previously performed QTL mapping studies to identify plasma lipid concentrations using mouse intercrosses [6–9]. We identified several significant QTL for plasma CHO and TG concentrations in C57BL/6J × DDD.Cg-*A*^*y*^ F_2_ female mice [9]. In the present study, we performed QTL mapping for plasma CHO and TG concentrations in F_2_ male mice produced via crosses between C57BL/6J and DDD/Sgn mice. Because male intercross population produced by these strains is not previously evaluated for plasma lipid concentrations, we expect novel QTL in addition to known QTL for plasma lipid concentrations to be identified. Furthermore, because there were apparent sex differences in plasma lipid concentrations between these mouse strains (e.g., female DDD.Cg-*A*^*y*^ mice had higher TG concentrations than males, whereas male C57BL/6J mice had higher TG concentrations than females [9]), we expect genetic aspects of sex differences to be revealed. Because men have a higher risk of coronary artery disease than women [10], it is crucially important to unravel the molecular basis of sex differences in plasma lipid concentrations.

## 2. Materials and Methods

### 2.1. Mice

The inbred mouse strains DDD and B6 were maintained at the National Institute of Agrobiological Sciences (NIAS, Tsukuba, Japan). Reciprocal crosses between DDD and B6 strains produced DB (♀DDD ×  ♂B6) F_1_ and BD (♀B6 ×  ♂DDD) F_1_ mice, both of which were intercrossed to produce DB F_2_ (*n* = 150) and BD F_2_ (*n* = 150) male mice [11].

All mice were weaned at 4 weeks of age and 4-5 mice were housed together in a cage during the experiments. All mice were maintained in a specific pathogen-free facility with a regular light cycle and controlled temperature and humidity. Food (CRF-1; Oriental Yeast Co., Ltd., Tokyo, Japan) and water were freely available throughout the experimental period. All animal procedures were reviewed and approved by the Institutional Animal Care and Use Committee of NIAS.

### 2.2. Plasma Lipid Analysis

Plasma lipid concentrations were determined at the age of 11 to 14 weeks in DDD, B6, and F_1_ mice and at the age of 11 to 12 weeks (71–80 days after birth) in F_2_ mice.

Mice were euthanized with an overdose of ether immediately after weighing in the morning. Blood was collected from the heart of an individual mouse using heparin as an anticoagulant. Plasma was separated by centrifugation at 2,000 ×g for 15 min at 4°C and was stored at −80°C until use. Plasma CHO and TG concentrations were determined by enzymatic methods using clinical chemical kits (Wako Pure Chemical Industries, Osaka, Japan).

### 2.3. Genotyping

Microsatellite sequence length polymorphisms were identified by electrophoresis after PCR amplification of genomic DNA. PCR amplification was carried out by use of a TaKaRa PCR Thermal Cycler Dice (TaKaRa Bio Inc., Shiga, Japan) under the following conditions: 1 cycle at 94°C for 3 min; 35 cycles at 94°C for 30 s, 55°C for 1 min, and 72°C for 45 s; 1 cycle at 72°C for 7 min. PCR products were separated on 10% polyacrylamide gel (Nacalai Tesque Inc., Kyoto, Japan) and were visualized by ethidium bromide (Nacalai Tesque) staining. A total of 117 microsatellite loci were genotyped. Their chromosomal positions were retrieved from MGI.

### 2.4. QTL Analysis

Normality of the trait data was assessed by Shapiro-Wilk* W* test (JMP8, SAS Institute Japan Inc., Tokyo, Japan). If the trait data did not follow a normal distribution, Box-Cox transformation was applied to the raw trait data (JMP8).

QTL mapping was performed using R/qtl version 1.38-4 [18, 19]. Single-QTL scans were performed by computing at 1 cM intervals across the entire genome using the cross direction (DB versus BD) as a covariate. Threshold logarithm of the odds (LOD) scores for significant (*P* < 0.05) and suggestive (*P* < 0.63) linkages were determined by performing 1,000 permutations [20]. After single-QTL scans, two-QTL scans were performed. In this case, we adhered strictly to the threshold recommended by Broman and Sen [18]. Finally, the combined effects of covariates and all QTL—including those that were significant and suggestive—were assessed using multiple QTL models [21].

### 2.5. Whole-Exome Sequence Analysis

Genomic DNA was extracted from the tail of DDD mice using a genomic DNA purification kit (Wizard Genomic DNA Purification Kit, Promega KK, Tokyo, Japan) and was submitted to Filgen, Inc. (Nagoya, Aichi, Japan) for exome capture and sequencing. Briefly, genomic DNA was subjected to the agarose gel and OD ratio tests to confirm the purity and concentration prior to Bioruptor (Diagenode, Inc., Denville, NJ, USA) fragmentation. Fragmented genomic DNAs were tested for size distribution and concentration using an Agilent Bioanalyzer 2100 and Nanodrop (Agilent Technologies, Wilmington, DE, USA). Illumina libraries were made from qualified fragmented genomic DNA using SPRIworks HT reagent kit (Beckman Coulter, Inc., Indianapolis, IN, USA), and the resulting libraries were subjected to exome enrichment using SureSelect XT Mouse All Exon Kit (Agilent Technologies) following the manufacturer's instructions. Enriched libraries were tested for enrichment by qPCR and for size distribution and concentration by an Agilent Bioanalyzer 2100. The samples were then sequenced on an Illumina HiSeq2000 (Illumina, San Diego, CA, USA), which generated paired-end reads of 90 or 100 nucleotides. Data was analyzed for data quality using FASTQC (Babraham Institute, Cambridge, UK). Sequence reads were mapped to the mouse reference genome (GRCm38, mm10). Read mapping and variant analyses were performed using CLC Genomics Workbench 7.0.4 and 8.5.1 (Filgen).

### 2.6. Statistical Analysis

Plasma lipid concentrations are represented as the mean ± SEM (mg/dL). Statistical differences between two groups were analyzed using Student's or Welch's* t*-tests. Tukey–Kramer honestly significant difference tests were used for statistical comparisons among more than two groups.* P* values < 0.05 were considered statistically significant.

## 3. Results

### 3.1. Plasma Lipid Concentrations in DDD, B6, and F_1_ Mice

Table 1 shows the statistical comparison of plasma CHO and TG concentrations between DDD and B6 mice and between DB F_1_ and BD F_1_ mice. Parental and F_1_ mice were analyzed separately. Among parental mice, both male and female DDD mice had significantly higher plasma lipid concentrations than their B6 counterparts. In F_1_ mice, excluding CHO concentrations in DB F_1_ and BD F_1_ males, both male and female DB F_1_ mice had significantly higher plasma lipid concentrations than their BD F_1_ counterparts. A clear lineage effect was thus observed.

### 3.2. Localization of Lipid QTL in DB F_2_ and BD F_2_ Males

**Figure 1:**
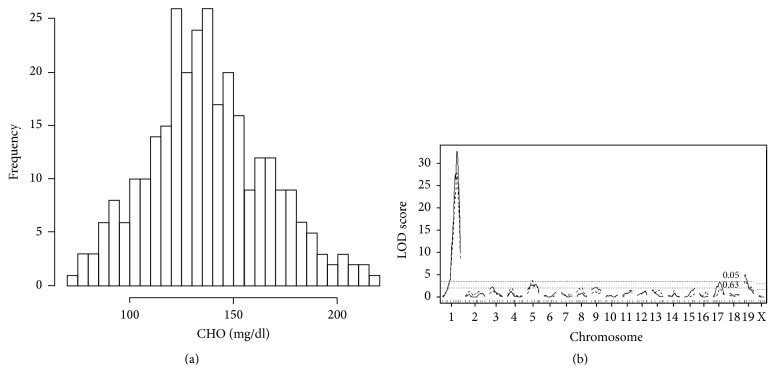
Genome-wide scan for plasma CHO concentrations in F_2_ male mice. (a) A histogram showing the distribution of plasma CHO concentrations. (b) Genome-wide LOD score plot of single-QTL scans for plasma CHO concentrations (solid lines: cross direction as an additive covariate; broken lines: cross direction and body weight as additive covariates). The *x*-axis represents the Chrs and microsatellite marker positions, and the *y*-axis represents the LOD score. The horizontal broken lines indicate the genome-wide threshold LOD score for significant (*P* < 0.05) and suggestive (*P* < 0.63) linkage, respectively.

**Figure 2:**
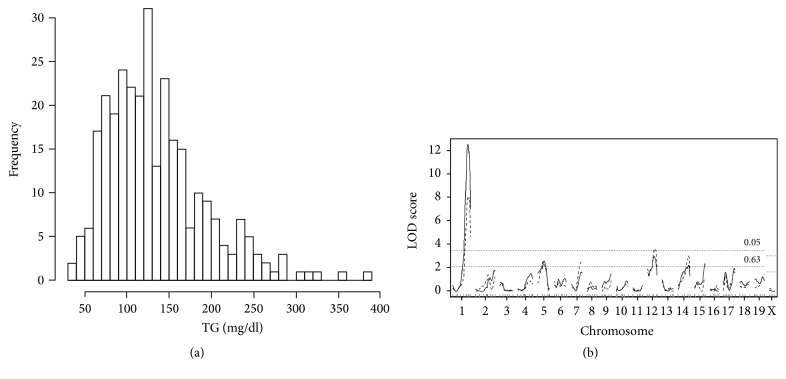
Genome-wide scan for plasma TG concentrations in F_2_ male mice. (a) A histogram showing the distribution of plasma TG concentrations. (b) Genome-wide LOD score plot of single-QTL scans for plasma TG concentrations (solid lines: cross direction as an additive covariate; broken lines: cross direction and body weight as additive covariates). The *x*-axis represents the Chrs and microsatellite marker positions, and the *y*-axis represents the LOD score. The horizontal broken lines indicate the genome-wide threshold LOD score for significant (*P* < 0.05) and suggestive (*P* < 0.63) linkage, respectively.

Histograms showing the distributions of plasma CHO and TG concentrations in 300 F_2_ males (data from 150 BD F_2_ and 150 DB F_2_ mice are combined) are presented in Figures 1 and 2. The distribution of CHO was bell-shaped (Figure 1(a)) but that of TG was slightly skewed (Figure 2(a)). Both traits were normalized after Box-Cox transformation (see Supplementary Figure  1 in the Supplementary Material available online at https://doi.org/10.1155/2017/3178204). For CHO, the difference between BD F_2_ (141 ± 2 mg/dL) and DB F_2_ (136 ± 2 mg/dL) males was not significant (*P* > 0.1). For TG, the difference between BD F_2_ (126 ± 5 mg/dL) and DB F_2_ (145 ± 5 mg/dL) males was significant (*P* < 0.002); therefore, cross direction (BD versus DB) was included as a covariate in subsequent analyses.

Genome-wide LOD score plots obtained via single-QTL scans for plasma CHO and TG concentrations in F_2_ males are shown in Figures 1 and 2. For CHO (Figure 1(b), solid lines), we identified three significant QTL on Chr 1@80.5 cM* (Choldq1)*, Chr 17@35.1 cM* (Choldq6)*, and Chr 19@8.0 cM* (Choldq7)* and three suggestive QTL on Chr 3@23.8 cM, Chr 5@59.8 cM, and Chr 9@37.0 cM (Table 2). The suggestive QTL on Chr 5 coincided with* Choldq4*, which was previously identified as a significant QTL in C57BL/6J × DDD.Cg-*A*^*y*^ F_2_ female populations [9]; therefore, we assigned the same gene symbol* Choldq4* to this QTL. For TG (Figure 2(b), solid lines), we identified one significant QTL on Chr 1@84.5 cM* (Trigdq1)* and four suggestive QTL on Chr 5@50.8 cM, Chr 12@47.0 cM, Chr 14@60.3 cM, and Chr 15@53.9 cM (Table 2). We also performed two-QTL scans to identify possible pairwise interactions between QTL but failed to identify significant interactions for both traits. Multiple-regression analyses indicated that the detected QTL explain 56.7 and 27.5% of the variations in plasma CHO and TG concentrations, respectively (Table 3).

There is a significant correlation between body weight and plasma lipid concentrations. That is, based on Spearman's rank correlation coefficient, the correlation between body weight and CHO concentration was 0.4855 (Spearman's *ρ*, *P* < 0.0001), the correlation between body weight and TG concentration was 0.4505 (Spearman's *ρ*, *P* < 0.0001), and the correlation between CHO and TG concentration was 0.4874 (Spearman's *ρ*, *P* < 0.0001). Therefore, we next performed single-QTL scans by including body weight together with cross direction as additive covariates. As a result, the abovementioned suggestive QTL for CHO on Chr 5 and suggestive QTL for TG on Chr 12 were identified as significant QTL (LOD score was 3.7 for both QTL) (Figures 1(b) and 2(b), broken lines). We named QTL on Chr 12* Trigdq2* (Table 2). We also performed two-QTL scans; however, there were again no significant pairwise interactions for both traits.

**Figure 3:**
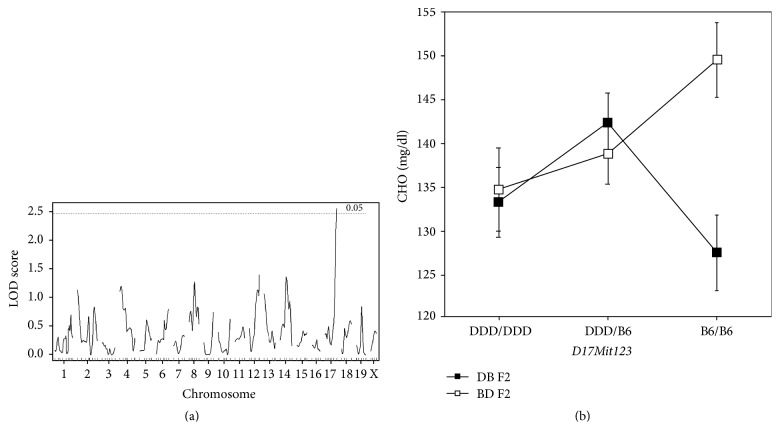
Genome-wide scan for QTL × cross direction interaction for plasma CHO concentrations. (a) Genome-wide LOD score plot. The *x*-axis represents the Chrs and microsatellite marker positions, and the *Y*-axis represents the LOD score. The genome-wide threshold LOD scores for a significant QTL × cross direction interaction were 2.4 for autosomes and 3.2 for Chr X, as indicated by horizontal broken line. (b) Allelic contributions of* Choldq8*, which interacts with cross direction. The *x*-axis shows the genotypes of F_2_ mice partitioned according to the nearest marker locus genotypes: homozygous DDD alleles are represented by DDD/DDD, homozygous B6 alleles are represented by B6/B6, and heterozygous alleles are represented by DDD/B6. The *y*-axis shows the average CHO concentrations, and the error bars are SEM.

We next searched for possible QTL that interact with cross direction (BD versus DB) by including cross direction as an interactive covariate. For CHO (but not TG), we identified significant QTL that interacted with cross direction on Chr 17@60.7 cM with LOD score 2.6 (threshold LOD score for significant QTL × covariate interaction was 2.4) (Figure 3(a)). Although the 95% confidence interval (CI) of this QTL (50.1–60.7 cM) slightly overlapped with that of* Choldq6* (17.1–51.1 cM), we assigned a new gene symbol,* Choldq8*, to this QTL, because the peak positions of the two QTL rather differed (*60.7* cM versus 35.1 cM). The allele effects of this QTL in DB F_2_ and BD F_2_ mice are shown in Figure 3(b) (although the CHO concentrations are shown in mg/dL, statistical analyses were done on transformed data; the allele effects of this QTL on transformed data are shown in Supplementary Figure 2).

### 3.3. Localization of Lipid QTL in F_2_ Males by Composite Interval Mapping

**Figure 4:**
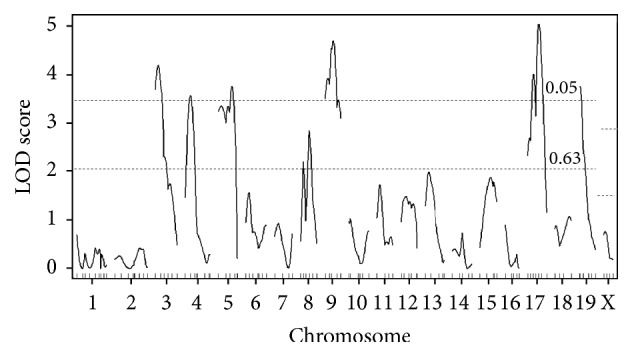
Genome-wide LOD score plot for CHO concentrations by composite interval mapping. The *x*-axis represents the Chr and microsatellite marker position, and the *y*-axis represents the LOD score. The horizontal broken lines indicate the genome-wide threshold LOD score for significant (*P* < 0.05) and suggestive (*P* < 0.63) linkage, respectively.

Because of the prominent phenotypic effect exerted by the Chr 1 QTL on both traits, we performed composite interval mapping by including the nearest marker for* Choldq1*/*Trigdq1*, that is,* D1Mit356*, and cross direction as additive covariates. Consequently, we identified six significant QTL for CHO, that is, Chr 3@19.8 cM* (Choldq9)*, Chr 4@23.1 cM* (Choldq10)*, Chr 5@59.8 cM* (Choldq4)*, Chr 9@37.0 cM* (Choldq11)*, Chr 17@37.1 cM* (Choldq6)*, and Chr 19@5.0 cM* (Choldq7)* (Figure 4 and Table 4). In contrast, we did not identify any significant QTL for TG.

### 3.4. Localization of Lipid QTL in Combined F_2_ Mice

**Figure 5:**
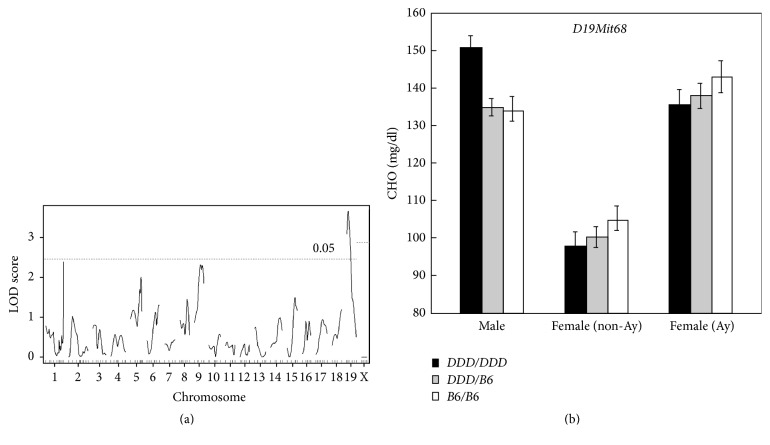
Genome-wide scan for QTL × sex interaction for plasma CHO concentrations. (a) Genome-wide LOD score plot. The *x*-axis represents the Chr and microsatellite marker position, and the *y*-axis represents the LOD score. The genome-wide threshold LOD scores for a significant QTL × sex interaction were 2.4 for autosomes and 2.9 for Chr X, as indicated by horizontal broken line. (b) Plasma CHO concentrations based on sex and the genotypes at* D19Mit68*. DDD/DDD denotes mice homozygous for the DDD allele, DDD/B6 denotes mice heterozygous for the DDD and B6 alleles, and B6/B6 denotes mice homozygous for the B6 allele. Error bars indicate SEM. In female mice, two kinds of F_2_ mice, that is, non-*A*^*y*^ (lean) and *A*^*y*^ (obese), according to the genotype at the* agouti* locus were analyzed [9].

Finally, we combined the data from this study on males with the previously analyzed data on B6 × DDD.Cg-*A*^*y*^ F_2_ females [9] and performed QTL mapping analysis. This analysis first aimed to increase the power of QTL mapping, as there was a possibility that we could identify additional and/or novel QTL and that the 95% CI for QTL could be narrowed by increasing the sample size. Second, we sought to identify possible QTL × sex interactions, as there may be QTL that significantly interact with sex. Such QTL would explain the difference in the results of QTL mapping performed between males and females. Because we analyzed two types of F_2_ mice, F_2_*A*^*y*^ and F_2_ non-*A*^*y*^, in our previous study of F_2_ females, trait data were standardized to a mean of 0 and a variance of 1 within each group of F_2_ males, F_2_  *A*^*y*^ females, and F_2_ non-*A*^*y*^ females, prior to analysis. As shown in Supplementary Figures 3A and 3B, standardized CHO followed a normal distribution but standardized TG did not. When sex was included as an additive covariate, we identified one additional significant QTL on Chr 11@61.4 cM* (Choldq12)* for CHO but none for TG (Table 5). We did not identify any additional significant QTL for TG by nonparametric interval mapping. Although the LOD scores for some QTL were increased, the 95% CIs for QTL were not necessarily narrowed sufficiently (e.g.,* Choldq1* on Chr 1). By including sex as an interactive covariate, we next searched for possible QTL × sex interactions for CHO. We identified one significant sex-interacting QTL on Chr 19@10.0 cM (LOD score: 3.6, 95% CI: 3.0–26.0 cM) (Figure 5(a)). This QTL coincided with* Choldq7*, which was identified by the single-QTL scan; therefore, we did not assign a new gene symbol to this QTL. The allele effects of this QTL in females and males are shown in Figure 5(b). The DDD allele tended to be associated with decreased CHO concentrations in females, whereas the DDD allele was significantly associated with increased CHO concentrations in males (although the CHO concentrations are shown in mg/dL, statistical analyses were done on standardized data; the allele effects of this QTL on standardized data are shown in Supplementary Figure 4).

### 3.5. Candidate Gene Search of Sex-Interacting QTL

We submitted the term “abnormal circulating cholesterol level” as a query to the MGI database (Mammalian Phenotype Browser), resulting in the retrieval of 1197 genotypes with 1905 annotations (MGI search was done on April 28, 2017). We consulted the MGI database (Genes and Markers Query Form) to determine the chromosomal localization of candidate genes. We performed whole-exome sequence analysis to identify nonsynonymous single-nucleotide variation (nsSNV), frameshift, and nonsense mutations as well as insertion-deletion (indel) in the coding regions of candidate genes in DDD mice. Of note, we found an Asp94Gly amino-acid substitution in* Acads* (caused by c.281A>G at Chr 5: 115,113,143) in DDD mice; this had been proposed as the nsSNV underlying the HDL-CHO QTL on Chr 5 [22] (see Discussion). We inspected genes on Chr 19 in particular because only a few QTL for relevant traits have been mapped to this chromosome. We found five candidate genes located within the 95% CI of* Choldq7* (*Lrp5*,* Pitpnm1*,* Naa40*,* Mark2*, and* Bscl2*) by MGI search but did not find any nsSNVs in any of these genes by whole-exome sequence analysis.

## 4. Discussion

This study identified a multifactorial basis for plasma lipid concentrations in F_2_ male mice generated by crosses between B6 and DDD inbred mice. We previously performed QTL mapping for body weight in the same F_2_ intercross population and identified four significant QTL on Chrs 1, 2, 5, and 17 [11]. Of these QTL, those on Chrs 1 and 5 coincided with QTL for CHO and TG concentrations, and that on Chr 17 coincided with QTL for CHO concentration on the basis of chromosomal localization (Table 2). Furthermore, the allele effect of each body weight QTL was in the same direction with that of lipid QTL. These results suggest a genetic link between body weight and plasma lipid concentrations. Indeed, we identified additional significant QTL,* Choldq4* and* Trigdq2*, by including body weight as an additive covariate. These QTL may have indirect effect on plasma lipid concentrations by acting through body weight. Because dyslipidemia and obesity often coincide with cardiovascular disease, knowledge of the genetic factors common to body weight and plasma lipid concentrations will be crucial for our understanding of the disease [13].

There was a clear lineage effect on plasma lipid concentrations between DB F_1_ and BD F_1_ males; that is, DB F_1_ mice had higher plasma lipids than BD F_1_ mice. Based on the studies of Y-consomic mice, the lineage effect was not due to influence by Chr Y [23, 24]. Furthermore, because we observed the lineage effect in the comparison between F_1_ females, we concluded that the effect of Chr Y on plasma lipid concentrations is extremely small or nonexistent. Because* Choldq8* (Chr 17@60.7 cM) interacts with cross direction, this QTL is involved in the observed lineage effect on CHO concentration. However, we cannot explain the lineage effect on TG concentration.

Most of the QTL identified in this study have coincidental QTL that had been reported by others [3, 4]. Above all,* Choldq1* on distal Chr 1 has numerous coincidental QTL for CHO and HDL-CHO concentrations. As observed for* Choldq1*, QTL identified in this region are known to have extremely large phenotypic effects [6, 8, 9]. The gene underlying these QTL is most likely* Apoa2*. When the crosses were composed of strains carrying different* Apoa2* alleles (where one strain must have an* Apoa2*^*b*^ allele), significant CHO QTL were invariably mapped to this chromosomal region [25]. Because DDD mice have the* Apoa2*^*b*^ allele and B6 mice have the* Apoa2*^*a*^ allele, it is unsurprising that a significant CHO QTL was mapped to this region. Thus,* Choldq1* is probably allelic with* Cq2*,* Cq6*, and* Hdlq20* [6, 8, 12].

*Choldq9* on Chr 3 was a coincidental QTL with* Cq3*, which was previously identified in B6 × KK.Cg-*A*^*y*^ F_2_ mice [6]. Both* Choldq9* and* Cq3* might be allelic because the B6 allele was associated with increased CHO concentrations in the two studies.

*Choldq4* on Chr 5 has at least two coincidental QTL for HDL-CHO, that is,* Hdlq1* (125 Mb) and* Hdlq8* (113 Mb) [22]. Su et al. [22] identified* Scarb1* as the gene underlying* Hdlq1* and* Acads* as the gene underlying* Hdlq8*. In particular, they demonstrated that the Asp94Gly amino-acid substitution in* Acads* was likely to alter the function of this protein because this change occurred in a highly conserved region. The Gly allele was associated with high HDL-CHO concentrations. DDD mice had the Gly allele, and B6 mice carried the Asp allele, suggesting that this was the nsSNV underlying* Choldq4*. Interestingly, we did not identify even a suggestive CHO QTL on Chr 5 in B6 × RR F_2_ mice [8], and both RR and B6 mice had the Asp allele (data not shown). Whereas Su et al. [22] reported they found no amino-acid changes in* Scarb1*, we identified one amino-acid change, Glu37Gln (caused by c. 109G>C at Chr 5: 125,304,335), in this protein. This amino-acid substitution was not identified in RR mice, in concordance with the absence of CHO QTL in B6 × RR F_2_ mice. Although the phenotypic effect of this amino-acid change is unclear,* Scarb1* may also be regarded as a potential candidate gene underlying* Choldq4*.

*Choldq11* on Chr 9 was a coincidental QTL with* Cq4* and* Cq5*, which were previously identified in B6 × KK.Cg-*A*^*y*^ F_2_ mice [6] and in KK × RR F_2_ mice [7], respectively. A proximal part of Chr 9 is known to have many genes relevant to lipid metabolism, including* Apoa1*,* Apoc3*,* Apoa4*,* Apoa5*, and* Lipc*, and multiple QTL including* Cq4*,* Cq5*, and* Hdlq17* [17] have been reported. We previously hypothesized that the lipid-associated QTL on Chr 9 were most likely due to polymorphisms in* Apoa4* [25]. We showed that a silent nucleotide change (c. 771C>T) in* Apoa4* was well correlated with the incidence of the CHO QTL. However, we did not identify this nucleotide change in DDD mice. Furthermore, we did not identify any amino-acid changes in* Apoa1*,* Apoc3*,* Apoa5*, and* Lipc* in DDD mice. Thus, we currently do not have any evidence that suggests that the above-mentioned genes are causative of* Choldq11*.

Based on their chromosomal localizations, two QTL have been reported as coincidental QTL with* Choldq6* on Chr 17. One was identified in an interspecific cross between CAST/Ei and B6 (no gene symbols were assigned) [26] and the other was* Hdlq29*, which was identified in an intercross population between NZB/BlNJ and B6 [12]. However, at variance with* Choldq6*, both QTL were identified only when the mice were fed on an atherogenic diet.

A relevant QTL mapped close to* Choldq7* on Chr 19 is* Hdlq48*, which was identified in an F_2_ intercross between B6 and A/J mice [14]. The peak position of* Hdlq48* was 4 cM with a 95% CI of 0–14 cM. However, at this QTL, the B6 allele was associated with higher HDL-CHO concentrations with an overdominant inheritance mode. These were apparently at variance with those of* Choldq7*, in which the B6 allele was associated with lower CHO concentrations with a dominant inheritance mode (Table 2). Another QTL,* Hdlq32* (peak position of 26 cM with a 95% CI of 10–70 cM), was also located on Chr 19 [12]. Although the 95% CI for* Hdlq32* overlapped with that for* Choldq7* (peak position of 7 cM with a 95% CI of 3–20 cM), the peak position differed. Furthermore, there were substantial differences between the two QTL; that is,* Hdlq32* was identified only when the mice were fed on a high-fat diet, and the phenotypic effect was observed only in females. Thus, there is no evidence supporting the possibility that both QTL are allelic. Thus,* Choldq7* on Chr 19 was suggested to be a novel QTL.

Because of its chromosomal localization,* Apoa2* appears to be the most suitable candidate gene underlying TG QTL* (Trigdq1)*. However, we do not believe this to be the case for the following reasons. All three inbred strains, namely, RR, DDD.Cg-*A*^*y*^ (DDD), and KK.Cg-*A*^*y*^ (KK), have the* Apoa2*^*b*^ allele. A significant TG QTL was identified in the* Apoa2* region in B6 × RR F_2_ female mice [8], B6 × DDD.Cg-*A*^*y*^ (DDD) F_2_ female mice [9], and the present F_2_ male mice but was not identified in B6 × KK.Cg-*A*^*y*^ (KK) F_2_ mice [6]. Thus, it appears that RR and DDD mice carry a different* Trigdq1* allele than KK mice, and we suspect that* Apoa2* is not the gene underlying* Trigdq1*. Several reports support this hypothesis; for example, Ishimori et al. performed QTL mapping for HDL-CHO and TG in F_2_ intercross populations between B6 and 129S1/SvImJ inbred mice and identified significant HDL-CHO QTL on distal Chr 1 but did not identify any TG QTL [13, 17]. Su et al. [27] performed QTL mapping for HDL-CHO and TG in F_2_ intercross populations between B6 and C3H/HeJ mice. They identified significant QTL for HDL-CHO but not for TG. Thus, the gene underlying the TG QTL identified on distal Chr 1 in mouse intercross populations is unlikely to be* Apoa2*.

Although numerous QTL for plasma lipid concentrations have been reported, there are additional QTL that remain to be identified. QTL analyses using a new strain combination will identify additional QTL. Identification of coincidental QTL will further substantiate the candidate genes underlying previously reported QTL.

## Supplementary Material

Supplementary Figures 1 and 3: Histograms of plasma CHO and TG concentrations. Supplementary Figure 2: Allelic contributions of *Choldq8*, which interacts with cross direction. Supplementary Figure 4: Allelic contributions of *Choldq7*, which interacts with sex.

## Figures and Tables

**Table 1:** Plasma lipid concentrations in DDD, B6, DB F_1_, and BD F_1_ mice.

Plasma lipids	Sex	Mean ± SEM plasma lipids (mg/dL)		*P* value	Mean ± SEM plasma lipids (mg/dL) (*n*)		*P* value
		DDD	B6		DB F_1_	BD F_1_	
CHO	Males	173 ± 4 (*n* = 38)	102 ± 5 (*n* = 21)	<0.0001	137 ± 4 (*n* = 15)	128 ± 5 (*n* = 12)	NS
	Females	162 ± 3 (*n* = 31)	92 ± 3 (*n* = 21)	<0.0001	119 ± 3 (*n* = 17)	110 ± 3 (*n* = 12)	<0.05
TG	Males	154 ± 11 (*n* = 38)	109 ± 15 (*n* = 21)	<0.03	200 ± 15 (*n* = 15)	112 ± 16 (*n* = 12)	<0.0005
	Females	194 ± 10 (*n* = 31)	43 ± 12 (*n* = 21)	<0.0001	108 ± 9 (*n* = 17)	65 ± 10 (*n* = 12)	<0.005

NS, not significant.

**Table 2:** Significant and suggestive QTL identified by genome-wide scans of F_2_ males.

Trait	QTL^a^	Chr	Peak cM	95% CI^b^	LOD^c^	Nearest marker	High strain^d^; inheritance^e^	Overlapping QTL	
								Name	Reference
CHO	*Choldq1*	1	80.5	77.5–85.5	**32.7**	*D1Mit356*	DDD, Add	*Cq2, Cq6, Hdlq20*	[6, 8, 12]
		3	23.8	10.8–56.8	2.3	*D3Mit25*	B6		
	*Choldq4*	5	59.8	17.8–75.8	2.9	*D5Mit239*	DDD	*Hdlq1, Hdlq8*	[13]
		9	37.0	12.0–59.6	2.2	*D9Mit207*	DDD		
	*Choldq6*	17	35.1	17.1–51.1	**3.4**	*D17Mit152*	B6, Add	*Hdlq29*	[12]
	*Choldq7*	19	8.0	3.0–19.0	**5.0**	*D19Mit68*	DDD, Rec	*Hdlq32, Hdlq48*	[12, 14]

TG	*Trigdq1*	1	84.5	77.5–93.5	**12.5**	*D1Mit356*	DDD, Dom	*Tgq3*	[8]
		5	50.8	17.8–66.8	2.6	*D5Mit239*	DDD		
	*Trigdq2*	12	47.0	13.0–62.0	2.9	*D12Mit259*	B6	*Tgq23*	[15]
		14	60.3	15.3–66.1	2.3	*D14Mit165*	DDD		
		15	53.9	40.8–53.9	2.4	*D15Mit193*	DDD		

QTL, quantitative trait loci; CI, confidence interval; LOD, logarithm of the odds.

Cross direction was included as an additive covariate in all analyses.

^a^QTL symbols, *Choldq4* and *Trigdq2* were assigned to suggestive QTL on Chrs 5 and 12, respectively, because they were identified as significant QTL if the body weight was included as an additive covariate. *Choldq4* was identified as significant QTL in our previous study in female mice [9].

^b^95% CI was defined by a 1.5-LOD decrease.

^c^LOD scores for significant QTL are indicated in bold. For CHO, the threshold LOD scores for significant and suggestive QTL were 3.4 and 2.1, respectively, for autosomes and 2.8 and 1.5, respectively, for Chr X. For TG, the threshold LOD scores for significant and suggestive QTL were 3.5 and 2.1, respectively, for autosomes and 2.8 and 1.5, respectively, for Chr X.

^d^High strain-derived allele was associated with higher plasma lipids.

^e^Mode of inheritance of high strain-derived allele. Dom, dominant; Add, additive; Rec, recessive.

**Table 3:** Multiple-regression analysis for plasma lipid concentrations.

Plasma lipid	Chromosome (cM)^a^	df^b^	Variance, %^c^	*F* value
CHO	Chr1@80.5	2	34.1	113.0
	Chr3@23.8	2	2.0	6.6
	Chr5@59.8	2	3.3	11.0
	Chr9@37.0	2	4.0	13.3
	Chr17@35.1	2	4.7	15.5
	Chr19@8.0	2	3.1	10.1
	Total	12	56.7	

TG	Chr1@84.5	2	15.1	30.1
	Chr5@50.8	2	2.0	4.0
	Chr12@47.0	2	2.7	5.4
	Chr14@60.3	2	2.5	5.1
	Chr15@53.9	2	1.6	3.2
	Total	10	27.5	

Cross direction was also included as a covariate.

^a^cM position on the chromosome.

^b^Degrees of freedom.

^c^Percentage of the total F_2_ phenotypic variance associated with each marker.

**Table 4:** Significant and suggestive QTL when *D1Mit356* was included as an additive covariate.

Trait	QTL^a^	Chr	Peak cM	95% CI^b^	LOD^c^	Nearest marker	High strain^d^; inheritance^e^	Overlapping QTL	
								Name	Reference
CHO	*Choldq9*	3	19.8	10.8–35.8	**4.2**	*D3Mit25*	B6, Add	*Cq3*	[6]
	*Choldq10*	4	23.1	9.1–37.1	**3.6**	*D4Mit286*	B6, Add	*Hdlq10*	[16]
	*Choldq4*	5	59.8	17.8–71.8	**3.7**	*D5Mit239*	DDD, Rec	*Hdlq1*, *Hdlq8*	[13]
	*Choldq2*	8	39.0	16.5–51.5	2.8	*D8Mit263*	B6		
	*Choldq11*	9	37.0	12.0–59.0	**4.7**	*D9Mit207*	DDD, Add	*Cq4*, *Cq5*, *Hdlq17*	[6, 7, 17]
	*Choldq6*	17	37.1	15.1–50.1	**5.0**	*D17Mit152*	B6, Rec	*Hdlq29*	[12]
	*Choldq7*	19	5.0	3.0–19.0	**3.7**	*D19Mit68*	DDD		

QTL, quantitative trait loci; CI, confidence interval; LOD, logarithm of the odds.

Cross direction was also included as an additive covariate.

^a^QTL symbols were assigned if they were significant or if they were suggestive but were identified as a significant QTL at least once previously in different genetic crosses.

^b^95% CI was defined by a 1.5-LOD decrease.

^c^LOD scores for significant QTL are indicated in bold. The threshold LOD scores for significant and suggestive QTL were 3.4 and 2.1, respectively, for autosomes and 2.8 and 1.5, respectively, for Chr X.

^d^High strain-derived allele was associated with higher plasma lipids.

^e^Mode of inheritance of high strain-derived allele. Add, additive; Rec, recessive.

**Table 5:** Significant and suggestive QTL for CHO identified by genome-wide scans of combined F_2_ mice (*n* = 598).

QTL^a^	Chr	Peak cM	95% CI^b^	LOD^c^	Nearest marker	High strain^d^
*Choldq1*	1	79.2	77.5–82.5	**64.7**	*Apoa2*	DDD
*Choldq9*	3	41.8	12.8–62.8	2.5	*D3Mit25*	B6
*Choldq4*	5	47.8	17.8–72.0	2.1	*D5Mit239*	DDD
*Choldq2*	8	21.2	14.5–53.5	**4.4**	*D8Mit191*	B6
*Choldq12*	11	61.4	34.4–75.4	**3.5**	*D11Mit124*	DDD
	12	60.6	23.0–62.2	2.5	*D12Mit141*	B6
*Choldq6*	17	19.7	13.1–49.1	3.3	*D17Mit176*	B6

QTL, quantitative trait loci; CI, confidence interval; LOD, logarithm of the odds.

Sex was included as an additive covariate.

^a^QTL symbols were assigned if they were significant or if they were suggestive but were identified as significant QTL at least once previously in different genetic crosses [9].

^b^95% CI was defined by a 1.5-LOD decrease.

^c^LOD scores for significant QTL are indicated in bold. The threshold LOD scores for significant and suggestive QTL were 3.5 and 2.1, respectively, for autosomes and 3.7 and 2.1, respectively, for Chr X.

^d^High strain-derived allele was associated with higher plasma CHO.
