# Supplementary material for: Identification of Quantitative Trait Loci That Determine Plasma Total-Cholesterol and Triglyceride Concentrations in DDD/Sgn and C57BL/6J Inbred Mice
Source: Cholesterol. 2017 May 31;2017:3178204. doi: 10.1155/2017/3178204 (PMC5469984; doi:10.1155/2017/3178204)

Legends to supplementary figures

Supplementary Figure 1. (A) A histogram showing the distribution of transformed plasma CHO concentrations. (B) A histogram showing the distribution of transformed plasma TG concentrations.

Supplementary Figure 2. Allelic contributions of *Choldq8*, which interacts with cross direction. The X-axis shows the genotypes of F_2 mice partitioned according to the nearest marker locus genotypes: homozygous DDD alleles are represented by DDD/DDD, homozygous B6 alleles are represented by B6/B6, and heterozygous alleles are represented by DDD/B6. The Y-axis shows the transformed average CHO concentrations, and the error bars are SEM.

Supplementary Figure 3. (A) A histogram showing the distribution of standardized plasma CHO concentrations. (B) A histogram showing the distribution of standardized plasma TG concentrations.

Supplementary Figure 4. Allelic contributions of *Choldq7*, which interacts with sex. The X-axis shows the genotypes of F_2 mice partitioned according to the nearest marker locus genotypes: homozygous DDD alleles are represented by DDD/DDD, homozygous B6 alleles are represented by B6/B6, and heterozygous alleles are represented by DDD/B6. The Y-axis shows the standardized average CHO concentrations, and the error bars are SEM. In female mice, data from two kinds of F_2 mice; i.e., non-A^y (lean) and A^y (obese), according to the genotype at the *agouti* locus were merged.

Supplementary Fig. 1A

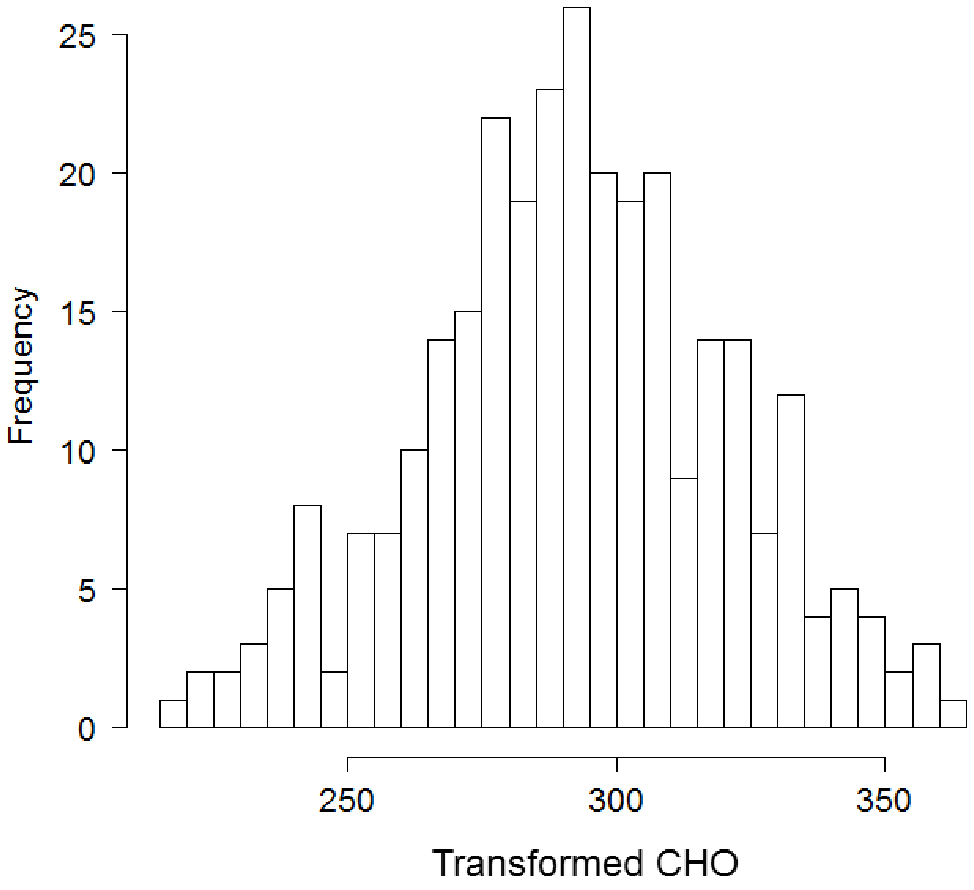

Supplementary Fig. 1B

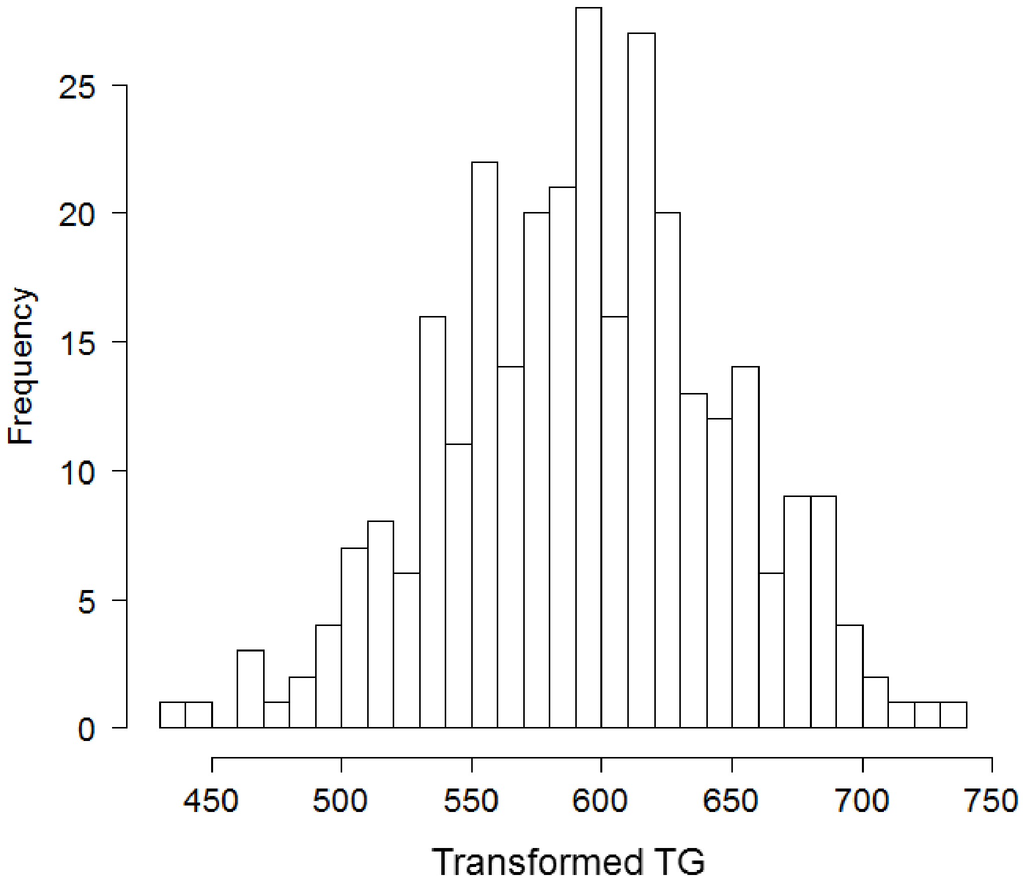

Supplementary Fig. 2

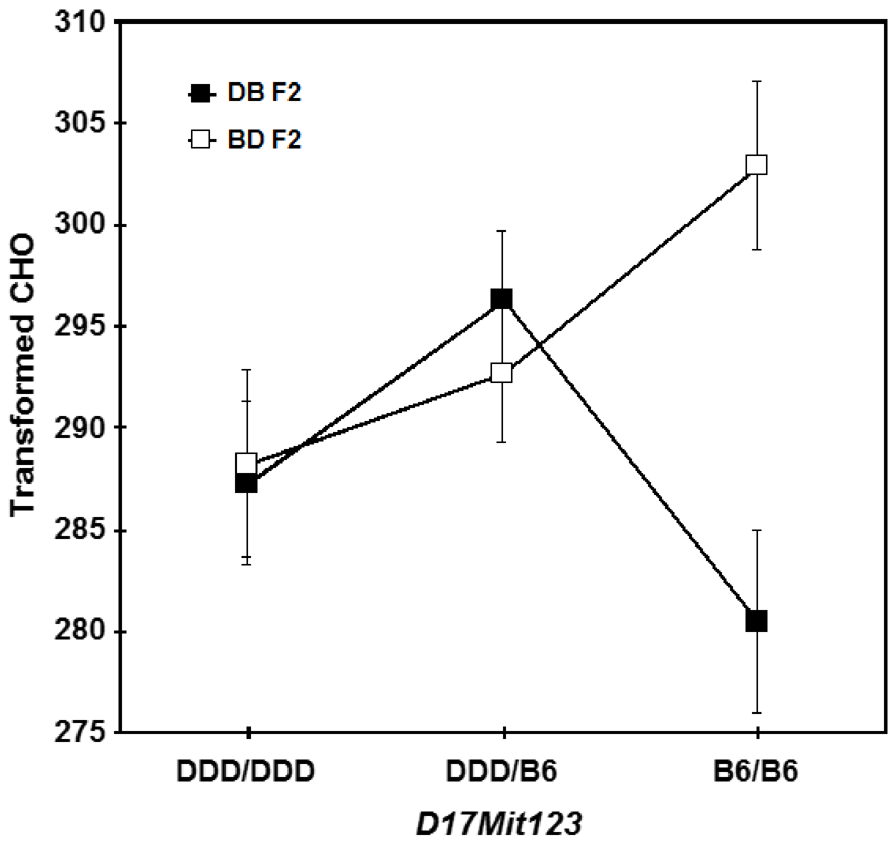

Supplementary Fig. 3A

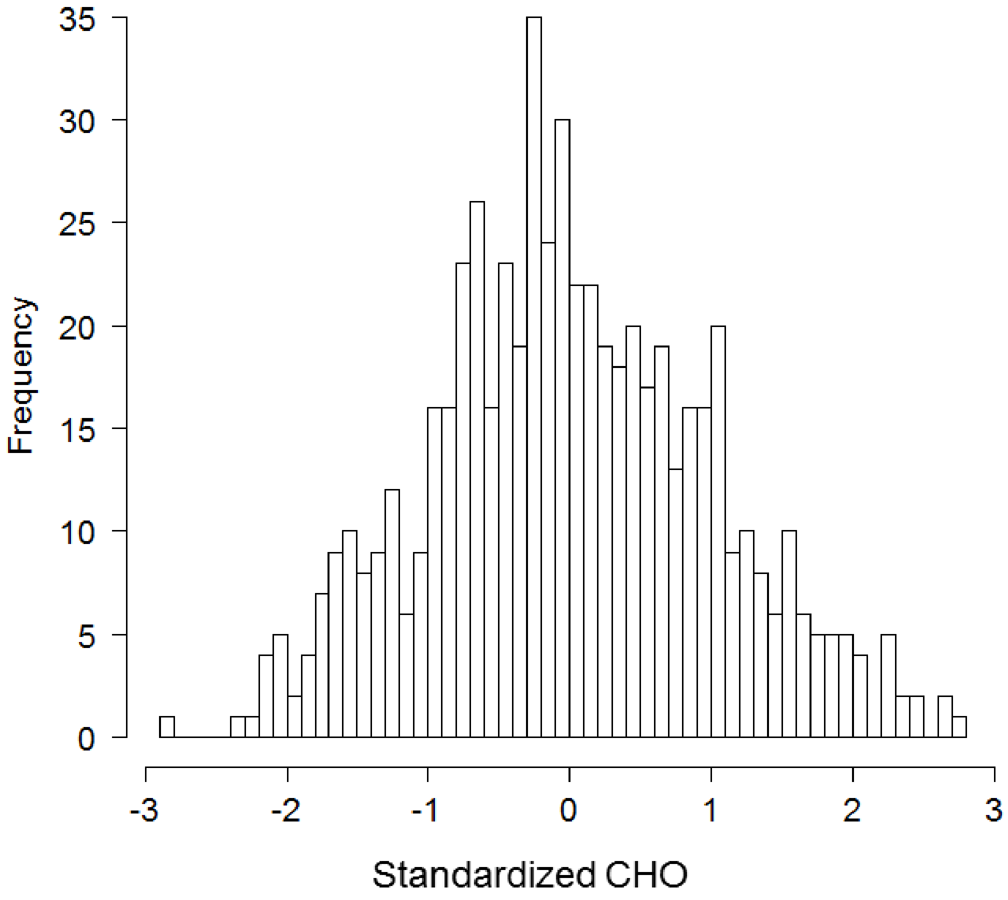

Supplementary Fig. 3B

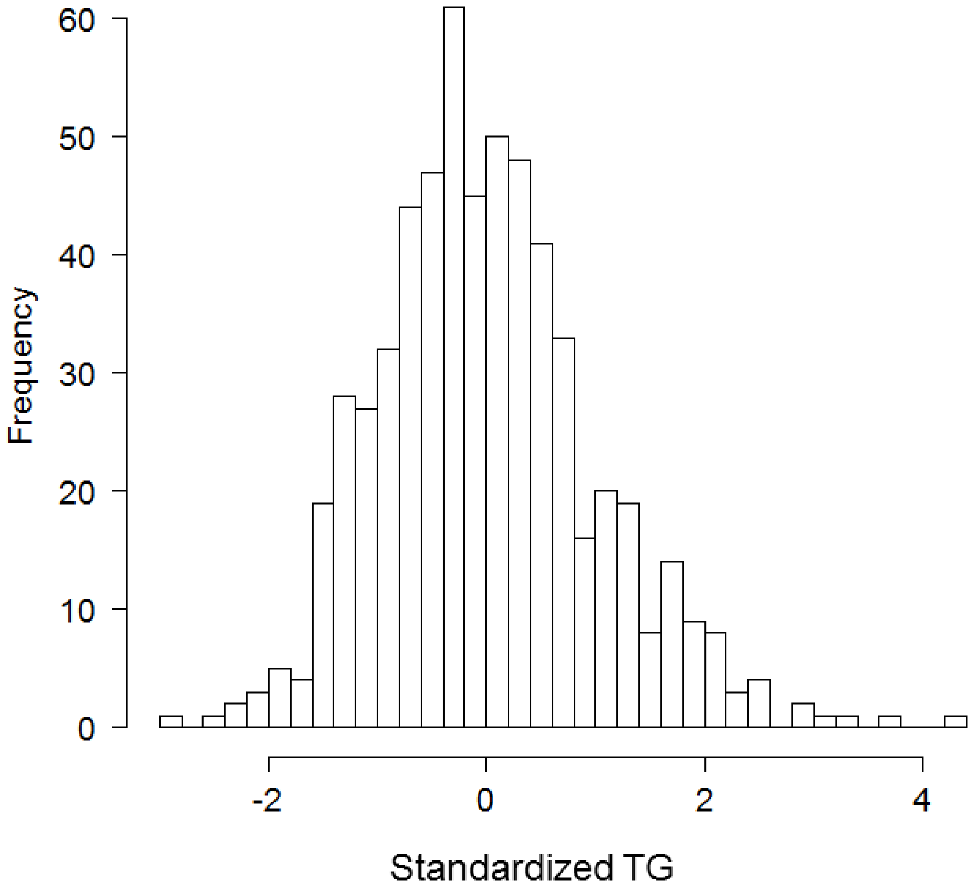

Supplementary Fig. 4

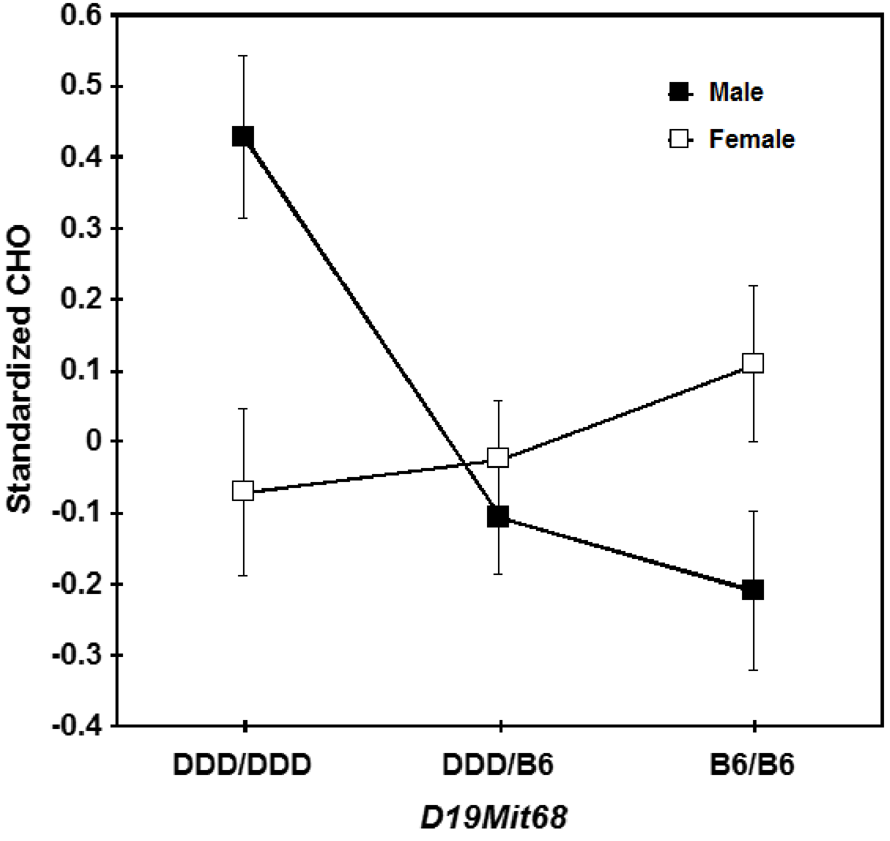

Supplement: Supplementary file 1 — Supplementary Figures 1 and 3: Histograms of plasma CHO and TG concentrations. Supplementary Figure 2: Allelic contributions of Choldq8, which interacts with cross direction. Supplementary Figure 4: Allelic contributions of Choldq7, which interacts with sex. [file 3178204.f1.pdf]
